# The Address in Medicine

**Published:** 1902-08-02

**Authors:** Thomas Barlow

**Affiliations:** Physician to His Majesty's Household; Physician to University College.


### THE ADDRESS IN MEDICINE.

By Sir Thomas Barlow, Bart., K.C.V.O., M.D., F.R.C.P., Physician to His Majesty's Household; Physician to University College.

In delivering the Address in Medicine, Sir Thomas Barlow took as his theme the general proposition that the study of the natural history of disease is the basis of all advance in its treatment. Notwithstanding the developments of science, and the large extent to which physicians have availed themselves of scientific methods, the fact remains that "the basis of our practice is empiricism. . . . Good empiricism continually strives to follow Nature's lead, and to shed that which is foolish and useless while preserving that which is essential; and by carefully recording cases, noting the results of changes made first by chance and then by design, good empiricism may at length assimilate to a truly inductive process, even though there are many unknown quantities. But as Nature herself seems sometimes to fail, and to leave behind vestiges and survivals which may lead to trouble, so we also in our treatment sometimes preserve methods of long-past utility which may now and again be harmful." Thus we owe much to the good sceptics who have arisen from time to time, who, throwing aside traditions and preconceptions "have taught us to look disease straight in the face with our own eyes, to realise what is its natural cycle, and to ask ourselves within what limits we can reasonably hope to modify it." It has been in the periods of half-knowledge that the practice of medicine has often cut a sorry figure, for theoretical lines of treatment, based on incomplete physiological and chemical data, have often produced melancholy failures. But the outcome of the new experimental pathology of the last quarter of a century, controlled by clinical observation, applied and tested by clinical experience, has justified us in claiming that at length our work has ceased to be fairly described as groping in the dark, and that in some definite instances medicine has justified for itself the designation of an exact science.

### Diphtheria.

In drawing attention to some recent notable advances in the healing art Sir Thomas Barlow maintained that the advances in the treatment of disease have been real and satisfactory in proportion as they have been harmonious with the complete knowledge of the natural history of disease. As a typical instance Sir Thomas Burton pointed to the inter-relation between the pathology and the treatment of diphtheria, recalling the chaotic and bewildering state of doctrine on the subject only a dozen years ago—the endless disputes as to the difference between croup and diphtheria, as to whether there was a true membranous croup which was different from laryngeal diphtheria, and as to the diagnostic differential signs of diphtheria. Malignant diphtheria of the throat was easy to diagnose, but as to the nomenclature of mild cases simulating follicular tonsillitis, and of the throat exudations complicating scarlet fever, an experienced man would be the last person to dogmatise. It was only at length by studying cases in epidemic groups, and especially by noting the paralytic sequelæ, that we learned how widely we had to cast our net in order to include examples in which there had been no visible membrane at all, though there might in some of them have been exudation in the post-nasal region, and, for a time, thin irritating discharge from the nostrils.

Then in regard to etiology, how diverse were our opinions. Exposure to cold was held to be a cause, and after cold came drains. Yet serious as is the effect of drain-poisoning in lowering vital resistance it has often been made to bear more than its fair share of blame in the causation of diphtheria, and sometimes the confident assurance on this doubtful point has been exceedingly mischievous, for though it led to sanitary investigations and helped sometimes to bring about sanitary improvements, it also led to the quiet ignoring of other possible sources of the disease. There was a time, not many years ago, when cases of membranous croup were treated in children's hospitals in ordinary wards, and there were those who stoutly maintained that these cases were not contagious. It was by careful study of the natural history of the disease that light came. It was seen that the disease was in some cases spread by milk, that epidemics were followed by sporadic cases or preceded by mild and ambiguous ones, that there was a relation between prevalence of the disease and compulsory school attendance, and finally that the disease was eminently contagious and was maintained and transmitted by the moving to and fro of slight cases.

### Antitoxin Treatment.

Again, think how varied were the lines of treatment. Who does not remember the routine administration of antimonials and emetics; the pitiful struggles of many a tender child at the swabbings which were fruitlessly and repeatedly applied; the continuous steam kettle and tent, and the irritating and headache producing antiseptic inhalations, by which we lowered the constitutional vigour of the patient while we vainly attacked the local disease? In regard to treatment also it was by that minute study of the natural history of the disease which we include under the term bacteriology, that light at last came. Passing over the details the solid gain to knowledge is that we now know that for the most part the mischief is elaborated locally at the site of the diphtheritic membrane, and not to any great extent by multiplication of the bacilli themselves in the blood and other tissues. And this leads us to see the justification of the researches of Behring and his fellow workers which eventuated in the practical application of the methods of immunisation and the preparation of an antitoxin.

The statistics as to the success which has followed the use of antitoxin are striking enough, for they show a reduction of mortality of between one-third and one-half. But statistics are not the only test upon which a doctor bases his practice. It is by the observation of actual cases that he becomes convinced that in antitoxin he has in his hands a remedy of enormous potency. When, within 48 hours, we see a young subject with malignant diphtheria accompanied by secondary infections which are on the verge of setting up septicæmia, so far relieved after the use of the remedy that the glands are reduced in size, the cellular tumefaction of the neck is lessened, the membranous exudation arrested and beginning to separate at the edges, the tongue more moist, and the general state ameliorated, we know that something has been done to control the disease processes with which no previous treatment we were acquainted with was in the least degree comparable. Proof of the same nature is also obtained by observing the progress of cases in which it has been necessary to perform tracheotomy. We see for ourselves that the membrane formation ceases to spread, and the tube, which has formidable disadvantages of its own, can be withdrawn much sooner, and the risks of ulceration and stenosis can be thereby lessened.

### Tuberculosis.

Turning now to the question of tuberculosis, it is the observation of the natural history of the malady that gives the key to much of our modern knowledge both as to the etiology and the treatment of the disease. The most important pathological fact bearing on the therapeutics of tuberculosis is the enormous frequency of unsuspected tubercle, the necropsies on people dying from surgical injury and every form of acute and chronic disease showing, in a great many cases, one or more old tuberculous lesions. The first reflection that forces itself upon us is the enormous frequency of unsuspected tuberculous illnesses that occur in the population around us. The second and equally important reflection is that there exists in the human subject a tendency to spontaneous limitation and arrest; and the third that there is an extreme frequency of local limited tuberculous deposit as compared with the generalised disease. How, then, are we to aid Nature in this spontaneous tendency to limitation and arrest?

Let us by no means deny the possible utility of serumtherapy, or of the tentative employment of such disinfecting measures as the use of formaldehyde according to the plan of Dr. Maguire, and let us be thankful for the means put at our disposal by surgeons and by those who employ the X-rays and Finsen's methods in the treatment of one or other form of tuberculous lesions; but surely the teaching of pathology, *i.e.*, of the observation of the natural history of the disease, is that the fortifying of the general resistance of the individual is the most important indication of all. Real true advance always tends to simplification, and the great fundamental therapeutic remedies are the employment of fresh air and sunshine, of good food, and of regulated exercises. This, surely, is the inwardness of what has been called the sanatorium treatment.

### Cancer.

And so one may go through other diseases and other modes of treatment, only to find illustrations of the fact that it is by the careful observation of the natural history of disease that advance in treatment is to be made. Even in regard to cancer, can there be any reasonable doubt that we are on the eve of a further elucidation of what has hitherto proved a mystery, and that some day the fragmentary and isolated pieces of knowledge which have been painfully and slowly acquired during past generations will slip into their places like the blocks in a puzzle of a child? But if the history of previous discoveries goes for anything this again will be an instance of the convergence of observation from many sides. Hence, it is our duty to do all that in us lies to maintain, to develop, and to defend experimental research in alliance with clinical work. "Our countrymen are wakening from their complacency, and are beginning to feel that in our great industries it is imperative, if we would keep our place, that well-directed scientific research should be liberally and steadily maintained; and shall it be said that in the care for the life and health of the community, the most precious of all industries, we are to be left crippled and poverty-stricken by the absence of suitable equipments?" For help in this matter we must not look entirely to the State. It is not that way that the genius of the English people lies, but rather to the municipality and to the generosity of Englishmen. "How much has been given for hospitals and charitable institutions, but how little for the endowment of research which will help to lengthen human life and mitigate its physical ills!"

